# Vulnerability Analysis and Passenger Source Prediction in Urban Rail Transit Networks

**DOI:** 10.1371/journal.pone.0080178

**Published:** 2013-11-18

**Authors:** Junjie Wang, Yishuai Li, Jingyu Liu, Kun He, Pu Wang

**Affiliations:** School of Traffic and Transportation Engineering, Central South University, Changsha, Hunan, P.R. China; Technical University of Madrid, Italy

## Abstract

Based on large-scale human mobility data collected in San Francisco and Boston, the morning peak urban rail transit (URT) ODs (origin-destination matrix) were estimated and the most vulnerable URT segments, those capable of causing the largest service interruptions, were identified. In both URT networks, a few highly vulnerable segments were observed. For this small group of vital segments, the impact of failure must be carefully evaluated. A bipartite URT usage network was developed and used to determine the inherent connections between urban rail transits and their passengers' travel demands. Although passengers' origins and destinations were easy to locate for a large number of URT segments, a few show very complicated spatial distributions. Based on the bipartite URT usage network, a new layer of the understanding of a URT segment's vulnerability can be achieved by taking the difficulty of addressing the failure of a given segment into account. Two proof-of-concept cases are described here: Possible transfer of passenger flow to the road network is here predicted in the cases of failures of two representative URT segments in San Francisco.

## Introduction

Faced with the rapid expansion of private vehicle ownership and use, which puts immense pressure on urban roads, transportation agencies in many cities have encouraged people to use public transportation in various ways [1]–[7]. Most large cities offer two types of public transportation. The first is urban rail transit (URT), which is characterized by high speed and large capacity. It usually forms the backbone of urban public transportation [8]–[13]. The other is conventional busing, which has considerable flexibility with respect to route planning and low operating costs. It extends the spatial coverage of public transportation services and improves the URT's fault tolerance by providing an alternative during breakdowns or scheduled maintenance events [8]–[10]. Urban rail transit can be considered the artery of modern public transportation. It transports a huge number of people in large cities, so its robustness and efficiency are of considerable importance and have drawn widespread attention in various scientific and engineering fields [8]–[12].

Using the statistical measures developed in complex network theory, researchers have studied the topologies and dynamics of urban rail transit networks [8]–[18]. Average path length, clustering coefficients, robustness, efficiency, passenger flow, and temporal evolution have been investigated to obtain deeper insights on how to improve public transportation environments [8]–[13]. Studies have shown that URT networks can have considerable efficiency and involve very low initial construction costs. However, their robustness can be low due to the lack of alternative paths between two stations (attributed to the expense of building rail tracks) [10]. Fortunately, during a URT failure conventional buses can offer URT passengers alternative routes along roads, markedly improving the URT network's fault tolerance. However, due to the lack of reliable data and appropriate methodologies, the spatial distributions of passengers' origins and destinations for each URT segment, which is crucial to coordinate operations between conventional buses and URTs, remains poorly understood.

In this paper, human mobility data from San Francisco and Boston were used to estimate morning peak travel demand and pinpoint the vulnerable segments of the San Francisco and Boston URT networks [19], [20]. A bipartite network framework is here proposed to predict the origins and destinations of passengers for each URT segment. This allowed high-quality measurement of the vulnerability of each URT segment. As a proof of concept of the proposed bipartite network, suitable bus routes capable of transporting URT passengers during the breakdown of two representative URT segments were here identified.

## Data and Methods

### URT networks

**Figure 1:**
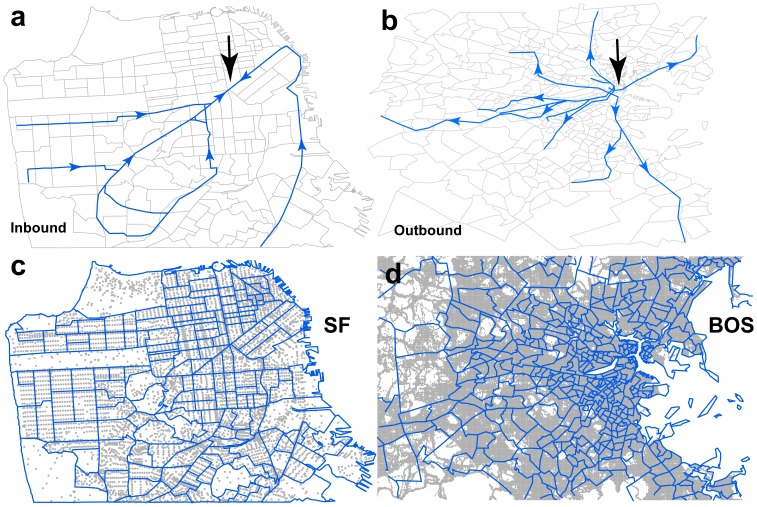
The URT network data and the human mobility data. (a) The black arrow points to the geographical center of the San Francisco URT network. The URT segments with directions heading to the geographical center are here considered “inbound” segments, and the URT segments with directions leaving away from the geographical center are considered “outbound” segments. (b) The geographic center and outbound segments in the Boston URT network. (c) The blue polygons on the San Francisco map indicate the census tracts, and the light gray dots indicate the centers of the city blocks. The San Francisco daily commuting OD data are recorded in a city-block resolution. (d) The blue polygons on the Boston map indicate the census tracts, and the light gray dots indicate the locations of mobile phone users detected during the three-week observational period.

Two URT systems are discussed in this paper. The San Francisco Muni Metro consists of 71.5 miles of track and six light rail lines and has an average weekday ridership of 173,500 passengers [21]. The Boston subway, called “the T,” is composed of three rapid transit lines and two light rail lines with an average weekday ridership of 628,400 passengers [22]. The coordinates of each station were collected using Google Earth, and maps of the URT networks were generated with nodes (123 in San Francisco, 122 in Boston) representing stations and links (254 in San Francisco, 246 in Boston) representing URT segments (Fig. 1a & b). The morning peak (8:00–9:00 a.m.) travel time of each link was estimated using official schedules [23], [24]. Geographical centers of the two URT networks were pinpointed and URT segments were classified as either inbound or outbound segments (Fig. 1a & b).

### Morning-peak URT ODs

Data regarding the daily commute in San Francisco were provided by the U.S. Census Bureau [25]. The number of commutes between each pair of city blocks was recorded (Fig. 1c). There are a total of 7,372 city blocks in San Francisco, and these data offered detailed information regarding where people live and work. The number of trips and daily home-to-work commute OD (origin-destination matrix) were calculated for residents who live and work in San Francisco. Commuting trips that started or ended outside the city were not considered. This was because the rate of usage of public transportation outside the city of San Francisco was very low and because public transport between cities is normally dominated by intercity rails.

Because daily commuting data for Boston were not available, large-scale mobile phone data were here used to estimate the home-work commute. In this scenario, each time a person uses his or her phone, the time of the call is recorded and the coordinates are estimated using a standard triangulation algorithm [26]. During the three-week observational period, more than 200,000 distinct locations were recorded (Fig. 1d). It was here assumed that a mobile phone user's home and work places were the locations where the user was most likely to be found from 9:00 p.m. to 6:00 a.m. and from 9:00 a.m. to 5:00 p.m. The user's commute was here considered the trip from his or her home location to his or her work location. Boston commuting OD was obtained by incorporating data covering all mobile phone users' commutes. Due to the data sampling bias introduced by the different penetration rates of mobile phones in different census tracts, the OD was adjusted using a down-scale exponent or an up-scale exponent 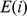 to render it more likely that the number of trips generated by a census tract would be proportional to its population:
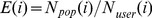
(1)


Here, 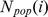 and 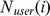 are the total population and the number of mobile phone users in census tract *i*. The number of commuting trips 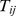 between locations *i* and *j* is calculated as follows:
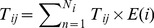
(2)


Here, 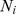 is the total number of users in census tract *i*. If the destination of user *n*'s commuting trip is location *j*, 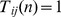, otherwise 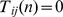.

People use different modes of transportation in their daily commute. These include driving, public transportation, and walking. Mode split data was here used to estimate the URT usage rate (modes included driving alone, carpooling, public transportation, walking, working from home, and other) [27]. It was here assumed that people who chose to walk were traveling distances under 1 km. For trips starting from census tract *i* and covering a distance larger than 1 km, public or private modes of transportation were assigned randomly according to the public transportation usage rate 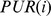 of that census tract (Fig. S1):
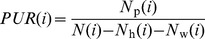
(3)


Here, 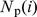 is the number of residents using public transportation in census tract *i*, 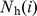 is the number of residents working from home, 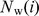 is the number of residents who walk to work, and 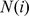 is the total population in the census tract. URT trips were extracted from the total number of trips involving public transportation. All of these were assumed to have origins and destinations within 500 meters of the URT stations. The origin and destination of each URT trip was connected to their nearest URT stations to generate the morning peak URT ODs. The San Francisco and Boston morning-peak hourly URT ODs included 13,005 and 33,500 trips, respectively. These values were found to be reasonably consistent with the average daily number of passengers who pass through Muni Metro and the T (Greater Boston subway system) [21], [22].

## Results

### Passenger flow in the URT networks

**Figure 2:**
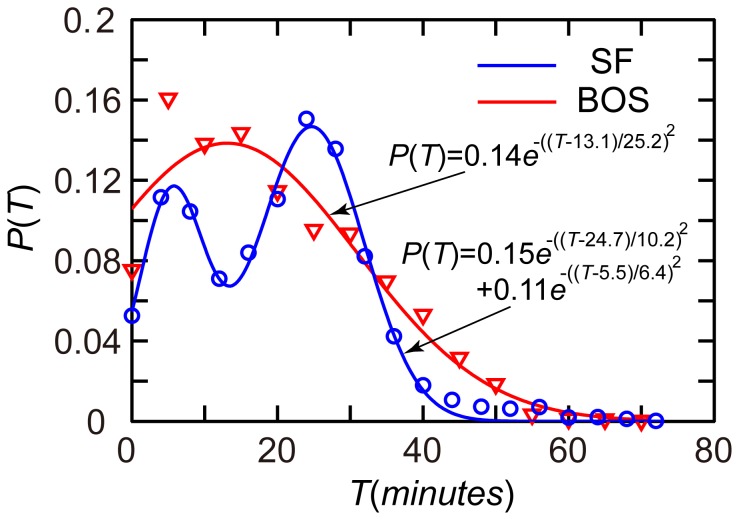
Distribution of the trip travel time. In San Francisco, the duration of a URT trip showed a double Gaussian distribution 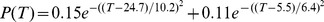
(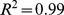). In Boston, the duration of a URT trip showed a Gaussian distribution 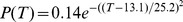 (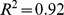).

The impact of the failure of a given URT segment was found to be closely correlated with its passenger flow. Based on the morning-peak hourly URT ODs, passenger flows in both URT networks can be estimated. In the present model, each passenger is assumed to use the shortest path, which is also assumed to involve the shortest travel time. The time cost with respect to waiting metro vehicles and transfers was not considered for purposes of simplicity. The shortest path for each URT trip was calculated using the Dijkstra algorithm [28]. Trip travel time was found to follow a double-Gaussian distribution in San Francisco with two peaks around 5 min and 25 min, and it was found to follow a Gaussian distribution in Boston with a typical time of roughly 10 min (Fig. 2).

**Figure 3:**
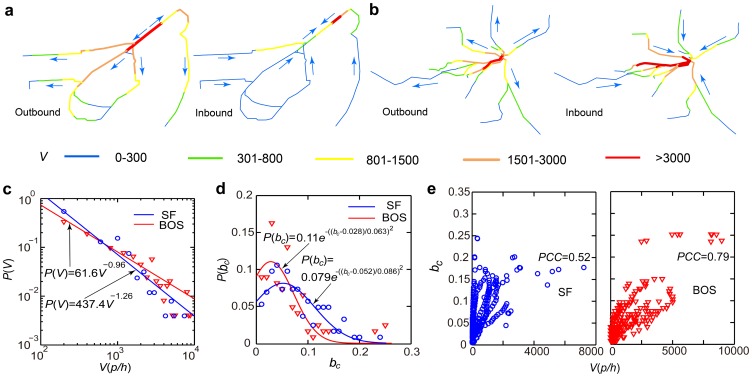
Passenger flow in the URT networks. (a) In the San Francisco URT network, the colors indicate the passenger flow of URT segment 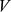. (b) Same as (a) but for the Boston URT network. (c) The passenger flow follows a power-law distribution 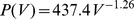 (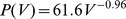) with 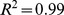 (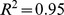) in San Francisco (Boston). (d) In San Francisco, the betweenness centrality 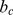 can be approximated by a Gaussian distribution 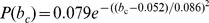
(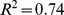). In Boston, the betweenness centrality 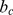 can also be approximated by a Gaussian distribution 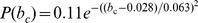 (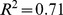). (e) Low correlations were observed between passenger flow 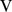
and the betweenness centrality 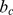. The topology of the Boston URT network was found to have a greater effect on shaping the passenger flow distribution than that of the San Francisco URT network did (*PCC* = 0.79).

Next, the passenger flow of each URT segment was measured by counting the number of shortest paths passing through each segment (Fig. 3a & b). Although 76% of the San Francisco URT segments showed passenger flow *V*<1,000 p/h (passengers/hour), the passenger flow of the busiest segment was found to reach 7,218 p/h. In San Francisco, passenger flow follows a power-law distribution 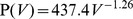, indicating that there may exist some extremely popular URT segments with very large passenger volumes (Fig. 3c). Similar heterogeneously distributed passenger flows can be observed in the Boston URT network, where the passenger flow was also found to follow a power-law distribution 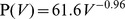 in which the maximum volume reached 8,970 p/h (Fig. 3c). Asymmetric URT usage patterns were also observed for inbound and outbound segments in both cities. In San Francisco, outbound URT segments showed larger passenger flows than inbound URT segments, but, in Boston, inbound segments showed larger passenger flows than outbound segments. The heterogeneously and asymmetrically distributed traffic flows indicated that failure of a few highly vulnerable segments could interrupt a large number of passengers.

In order to indicate the importance of the URT ODs to the prediction of passenger flow, betweenness centrality 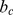, which only quantifies the topological importance of a link, was also measured for each URT segment [29]–[31]:
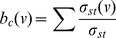
(4)where 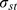 is the number of shortest paths from node 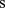 to node 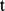 and 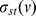 is the number of shortest paths passing through segment 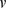. Fig. 3d shows that the URT segments' betweenness centrality can be approximated by Gaussian distributions in both San Francisco and Boston:
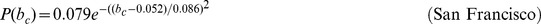
(San\ Francisco)\ (5)

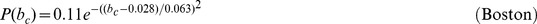
(Boston)\ (6)


Unlike passenger flows (power-law distributions with fat tails), typical values of betweenness centrality 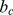 ∼0.05 and 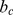 ∼0.03 can be found in the two URT networks, suggesting that the usage of the URT networks is shaped by actual travel demands (URT ODs). The Pearson correlation coefficient (*PCC*) between passenger flow 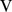 and betweenness centrality 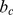 can be measured for San Francisco (*PCC* = 0.52) and Boston (*PCC* = 0.79), again confirming that the URT ODs are necessary to estimate passenger flow accurately (Fig. 3e).

### Vulnerable segments within the URT networks

**Figure 4:**
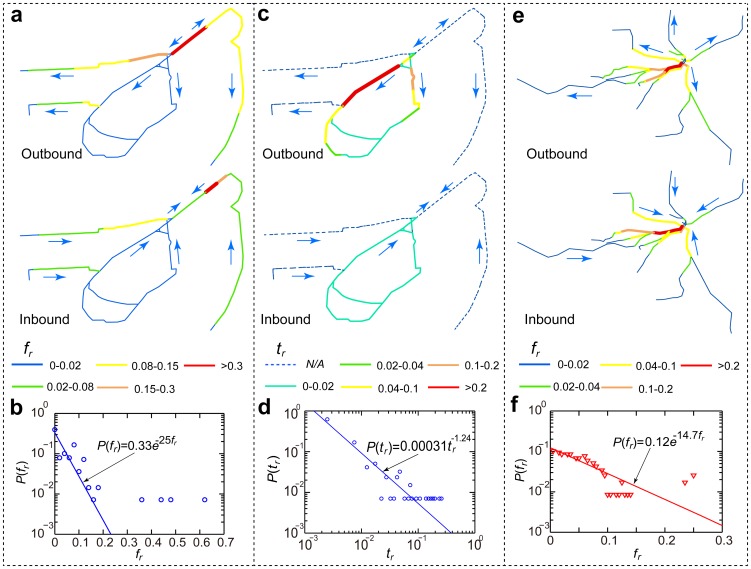
Vulnerable URT segments in the URT networks. (a) In the San Francisco URT network, the color of each URT segment represents the relative number of failed trips 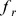. (b) The trip failure rate 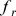 can be approximated using an exponential distribution 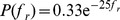
(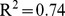) in San Francisco. (c) The color of a San Francisco URT segment represents its trip time increasing rate 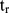. Only the segments in the loops subject to trip time increase. A maximal 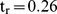 is observed. (d) The rate of increase of trip time showed a power-law distribution 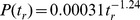 (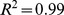). (e) In the Boston URT network, the color of each URT segment represents the relative number of failed trips 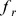. (f) The trip failure rate 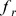 can be approximated by an exponential distribution 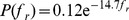 (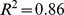) in Boston.

The passenger flows for the two URT networks were calculated using the Dijkstra algorithm. Each URT segment's vulnerability could then be calculated by defining the trip failure rate 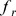, which is here the relative number of trips that fail in the specific segment's break down. Measuring the trip failure rate 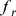 for each URT segment, produces a comprehensive picture of the locations of the vulnerable parts of the URT networks. Roughly 67% of the segments in the San Francisco URT network showed a trip failure rate 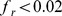, but the trip failure rates 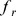 for a small number (2.8%) of high-volume segments were over 0.15 (Fig. 4a). The highest trip failure rate in San Francisco was found to be 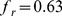, meaning that 63% of the URT trips will be interrupted in the failure of the segment. The trip failure rate 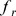 of the San Francisco URT network showed an exponential distribution 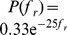 (Fig. 4b), showing a spatial distribution similar to that of passenger flow except for the segments in the loops. Alternative URT routes were observed when the segments in the loops broke down. In this way, the trip failure rate for these segments was equal to zero. However, the failure of these URT segments can increase travel time through time-consuming detours. To quantify this effect, the rate of increase in trip time, 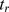, of each URT segment was defined as the relative amount of the increase in time required for an average trip in the event of segment breakdown. The rate of increase in trip time, 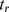, can approximated closely using a power-law distribution 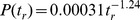 (Fig. 4c & d). The maximum 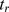 was observed at 0.26, meaning that average travel time increases 26% when the segment in question breaks down. This is a tremendous decrease in efficiency for the URT network.

The exponentially distributed trip failure rate of the Boston URT network, 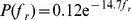
was determined here (Fig. 4e& f). The trip failure rate was under 0.02 for 51.6% of the URT segments. The largest trip failure rate was found to be 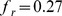, indicating again that some segments are highly vulnerable segments, whose breakdown would result in the failure of a large number of trips.

### Bipartite networks of URT usage

One important issue faced by transportation agencies during a URT failure is acquiring information on the individuals affected by it and on their origins and destinations. This information can be extracted by generating a bipartite network of URT usage, which pinpoints the major sources of passengers and the major destinations of the individuals who use each URT segment. Bipartite networks are a particular class of complex networks whose nodes are divided into two sets, and connections are only allowed between two nodes in different sets [32]. To generate the bipartite network of URT usage, the URT segments can be grouped into one set of nodes, and the census tracts that produce and attract passengers can be grouped into the other set of nodes.

**Figure 5:**
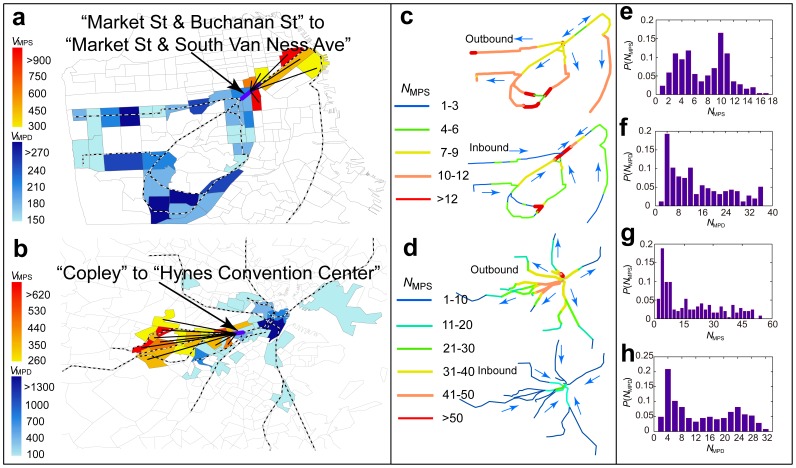
Locating major passenger sources (MPS) and major passenger destination (MPD). (a) In the San Francisco URT network, the census tracts in yellow, orange, and red indicate the MPS passenger production values of the most vulnerable segment. The census tracts shown in shades of blue represent the segments' MPD passenger attraction values. Black links indicate the connections between each selected URT segment and its MPS. (b) In the Boston URT network, the census tracts in yellow, orange, and red indicate the MPS values of the most vulnerable segment. The census tracts shown in shades of blue represent the segments' MPD values. Black links indicate the connections between each selected URT segment and its MPS. (c) The color of each segment represents its number of MPS (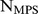). (d) The color of each segment represents its number of MPS (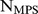). (e) The distribution of the number of major sources of passengers 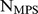 in San Francisco. (f) The distribution of the number of major passenger destinations 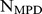 in San Francisco. (g) The distribution of the number of major sources of passengers 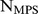 in Boston. (h) The distribution of the number of major passenger destinations 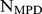 in Boston.

The most essential part of generating the bipartite URT usage network is connecting the two sets of nodes. For each passenger, the census tracts in which his or her home and workplace are located must be pinpointed and the URT segments that he or she uses must be predicted. For a URT segment, each passenger's home census tract is counted once if his or her trip passes through the segment. By incorporating all passenger trips that pass through the URT segment, the passenger flows contributed by each census tract can be quantified and ranked (passenger source). To locate the major sources of passenger flow, a URT segment's major sources of passengers (MPS) were here defined as the top ranked census tracts from which 80% of its passenger flow originated. Linking each URT segment (belonging to one set of nodes) to its MPS (belonging to the other set of nodes), facilitated the generation of the bipartite network of URT usage, where the degree of a URT segment is the number of its MPS (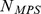) and the degree of a census tract is the number of segments for which it serves as the MPS (Fig. 5a & b). Similarly, we define a URT segment's major passenger destinations (MPD) as the top ranked census tracts that attract 80% of its passenger flow. The number of major passenger destinations is then denoted as 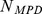. Connecting each URT segment with its MPD we generate another bipartite network of URT usage, which pin points the major passenger destinations of each URT segment. As Fig. 5a & b shows, we pinpoint the MPS and the MPD for the URT segments with the highest trip failure rate 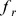 in San Francisco and Boston. The sources and destinations of the passengers who are most severely affected by failure of the indicated segments can be located easily using the bipartite networks of URT usage.

Next, the number of major passenger sources and the number of major passenger destinations was determined for each URT segment. Two groups of URT segments with 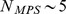 and 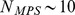 can be identified in San Francisco (Fig. 5c & e). Nearly 90% of the URT segments have fewer than 12 major sources of passengers, suggesting that sources of passengers can be located easily. The largest 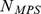 observed in San Francisco was only 17, which was much smaller than the maximum number of major passenger destinations (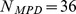). This suggested that employees at many workplaces are concentrated in relatively few residential areas (Fig. 5f). The Boston URT segments can be classified into two groups using their 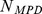. These two groups of segments showed 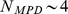 and 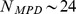, respectively (Fig. 5h). Boston showed a larger maximum 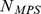 than San Francisco, 54, but it had a smaller maximum 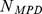, 32. This suggested that employees at many residential areas are concentrated in relatively few work places (Fig. 5d &g). This difference could be caused by differences in land use patterns in these two cities [1].

### A new aspect to the measurement of the vulnerability of URT segments

Given that 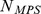 and 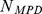 can characterize a URT segment's role in a URT network and illustrate its association with passenger diversity, a new quality in the understanding of URT segments' vulnerability can be achieved by combining 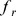 with 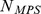 and 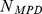. The URT segments with large 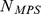 and 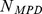 values have more widely distributed sources of passengers and possible destinations, so breakdowns of these segments will pose more substantial challenges to transportation agencies. If two URT segments have the same trip failure rate, 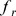, then failure of the one with the larger 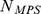 or 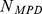 value will involve the interruption of a more complicated passenger flows, increasing the difficulty of offering appropriate bus routes.

The URT segments were grouped according to their 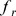, 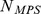 and 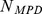 values. URT segments with 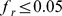 and 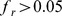 were here defined as minimally vulnerable segments and highly vulnerable segments, respectively. URT segments with 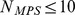 (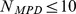) and 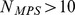 (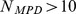) were defined as low-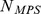 (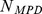) segments and high-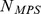 (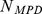) segments. In both URT networks, the number of major sources of passengers, 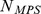, and the number of major passenger destinations, 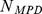, were found to be minimally correlated with the trip failure rate 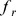. The URT segments can be classified into four groups according to their 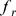 and 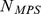 (Fig. S2a & b). Red symbols represent the segments with high 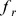 and high 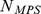, blue symbols represent segments with high 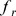 and low 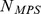, green symbols represent segments with low 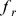 and high 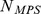, and gray symbols represent segments with low 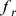 and low 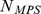. Similarly, URT segments can be classified into four groups according to their 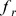 and 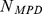 values (Fig. S2c and d). The spatial distributions of the eight groups of URT segments are depicted in Fig. S3. The segments shown in red are the most vulnerable, which here means that failure of these segments would have the highest impact and that these events would be the most difficult to handle. Segments shown in blue are also vulnerable in that failure would have a high impact, but the difficulty of responding to these events would be much less pronounced. Incorporating the proposed parameters 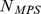 and 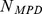 into the estimation of a URT segment's vulnerability allows the difficulty of handling these failure events to be taken into account.

In addition to the eight groups of URT segments classified by their 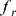, 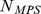 and 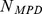, two clusters of nodes naturally appeared in the upper and lower arcs (Fig. S2). These two clusters were found to correspond to the inbound and outbound URT segments, respectively. This clustering pattern can be explained by the two components that determine a URT segment's 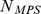 and 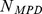: topological location and the passenger flow. In the URT networks studied here, the routes passing through an outbound segment can originate from stations in several URT lines (due to transfers) but they usually end at stations in one line. In contrast, the sources of trips passing through an inbound segment are usually limited to one URT line, but their destinations may fall along many lines. In this way, in the URT networks studied here, the inbound segments tended to show larger 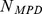 values, and the outbound segments usually showed larger 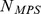 values. However, it is not necessarily the case that an inbound segment always has larger 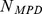 than an outbound segment or that an outbound segment always has larger 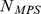 than an inbound segment. URT segments with high 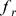 values tended to have larger 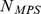 and 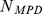 values, producing the clustering patterns shown in Fig. S2. In conclusion, the 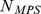 and 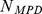 of a URT segment depend not only on its topological location but also on its passenger flow, which is closely correlated with the trip failure rate 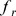.

### Proof of concept use of the bipartite network of URT usage

**Figure 6:**
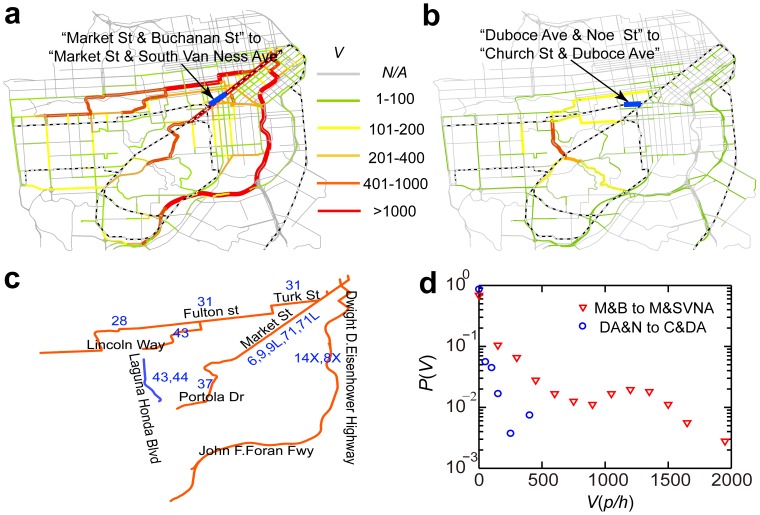
Alternative routes in the road network. (a) The color of a road segment represents the potential passenger flows transferring to it when the URT segment from Market St. and Buchanan St. to Market St. and South Van Ness Ave. fails. (b) Same as (a) but for an actual disconnection of the URT segment from Duboce Ave. and Noe St. to Church St. and Duboce Ave. during a maintenance project in San Francisco. (c) The potential alternative bus routes when the URT segment from Market St. and Buchanan St. to Market St. and South Van Ness Ave. breaks down (red lines) and the potential alternative bus routes that were available during the maintenance project (blue lines). (d) The distribution of passenger flow indicates different patterns of travel demand put on the San Francisco road network.

The importance of the bipartite URT usage network also relies on its ability to facilitate the sharing of information between different transportation systems. As a proof-of-concept, the URT segment stretching from Market Street and Buchanan Street to Market Street and South Van Ness Avenue, which has the highest trip failure rate 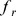 of any segment evaluated in the present work, may serve as an example. The transfer of passenger flow to the San Francisco road network in the event of a breakdown of this URT segment can be calculated. Passenger origins and destinations were extracted for URT trips starting at any of this segment's major sources of passengers. By connecting each origin and destination to its nearest road intersection, the shortest paths for each selected trip in the road network were determined [33], [34]. The numbers of passengers transferred to the road network ranged from 0 to 2,100. Three major routes of passenger flows are depicted in Fig. 6a. The roads along the major routes include Market Street, Lincoln Way, and the Bayshore Freeway. Buses 14X, 6, 31, and a few others have services in these road sand can provide alternative paths for passengers whose trips are interrupted by the failure of the URT segments in question (Fig. 6c). Another example involves an actual disconnection of the San Francisco URT segment from Duboce Avenue and Noe Street to Church Street and Duboce Avenue, which is reported by the Municipal Transportation Agency as “Church and Duboce Track and Street Improvement Project.” Possible transfers of passenger flow to the road network during this maintenance project can be calculated (Fig. 6b). Laguna Honda Boulevard (highlighted in red) is here predicted to see the most intensive use during this period. Buses 44 and 43 have service on this road (Fig. 6c). The distributions of the possible passenger flows induced in the road network show different patterns in upper two examples (Fig. 6d), suggesting that different strategies need to be implemented in dealing with the emergent events taking place in the two URT segments. In conclusion, the bipartite network of URT usage can offer a feasible modeling framework for conducting effective counter measures against possible failures in URT networks.

## Discussion

Estimation of the morning peak commuting ODs was used to locate the vulnerable segments in the San Francisco and Boston URT networks. The unevenly distributed vulnerability of the two networks suggests that special care must be taken for those highly vulnerable segments. By incorporating the proposed new measures, 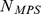 and 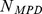, into the estimation of vulnerability, not only was the impact of a URT segment's failure captured but the issues related to addressing difficulty were taken into account. In this way, a new layer of the vulnerability of URT segments was here described. The proposed bipartite network of URT usage offers a useful modeling framework that can be used to locate the origins and destinations of passengers who use the most vulnerable segments of the network and to address emergencies by providing alternative services. More dedicated and detailed data, such as the subway card data, which would include information regarding where people enter and exit the URT, could be included in the current modeling framework, which would render it more accurate. A framework that could be used to predict transfers of passenger flow from the URT network to the road network during failure of two representative URT segments is here proposed. This may provide insight into the optimization of the reliability and efficiency of multi-layer public transportation systems [35], [36]. Information sharing techniques can be used to transfer messages between different layers of a public transportation system. This would improve the system and so encourage more people to use public transportation.

## Supporting Information

Figure S1 Public transportation usage rates in San Francisco and Boston. The mode split data were collected using TransCAD [27]. The numbers of residents using different modes of transportation (driving alone, carpooling, public transportation, walking, working from home, and other) were recorded for each census tract.
(TIF)

Figure S2Correlations among 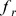, 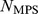, and 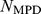. (a) Four groups of URT segments were classified according to their 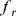 and  values. Red symbols represent the highly vulnerable segments (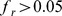) which also showed large numbers of major passenger sources 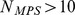. Blue symbols represent URT segments with high 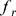 and low 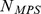 values, green symbols represent those with low 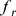 and high 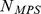 values, and gray symbols represent those with low 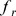 and low 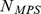 values. Circles represent the inbound URT segments, and triangles represent outbound URT segments. (b) The same classification system and symbols were used for Boston. (c) Red symbols represent URT segments with high 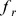 and high 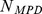 values, Blue symbols represent URT segments with high 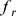 and low 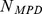 values, green symbols represent those with low 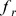 and high 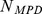 values, and gray symbols represent those with low 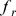 and low 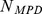 values. (d) See (c).(TIF)

Figure S3Spatial distributions of the eight groups of URT segments defined by 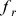, 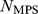, and 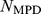. (a) Spatial distribution of the four groups of San Francisco URT segments classified using to their 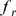 and 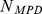
values. (b) The spatial distributions of the four groups of San Francisco URT segments were classified according to their 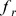 and 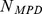 values. (c) Spatial distribution of the four groups of Boston URT segments classified using to their 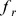
and 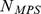 values. (d) The spatial distributions of the four groups of Boston URT segments were classified according to their 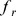 and 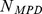 values.(TIF)
