# Supplementary figures and images for: Vulnerability Analysis and Passenger Source Prediction in Urban Rail Transit Networks
Source: PLoS One. 2013 Nov 18;8(11):e80178. doi: 10.1371/journal.pone.0080178 (PMC3832441; doi:10.1371/journal.pone.0080178)

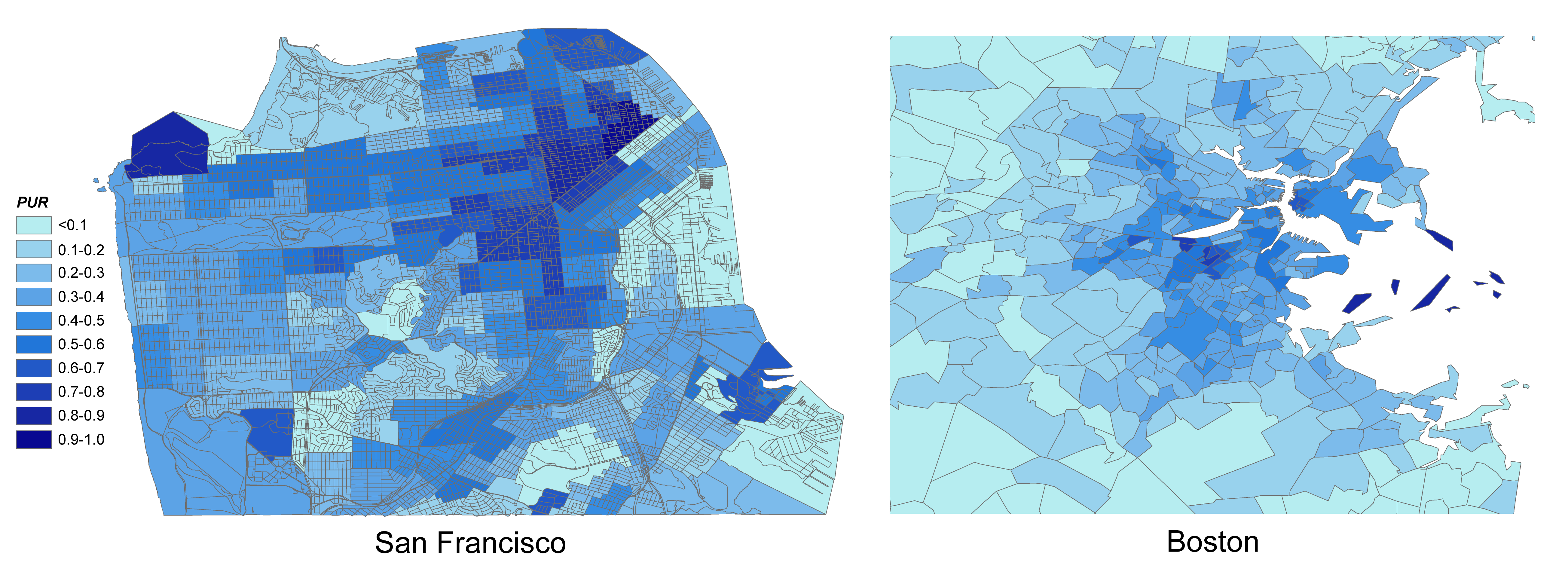

Supplement: Figure S1 — Public transportation usage rates in San Francisco and Boston. The mode split data were collected using TransCAD [27]. The numbers of residents using different modes of transportation (driving alone, carpooling, public transportation, walking, working from home, and other) were recorded for each census tract. (TIF) [file pone.0080178.s001.tif]

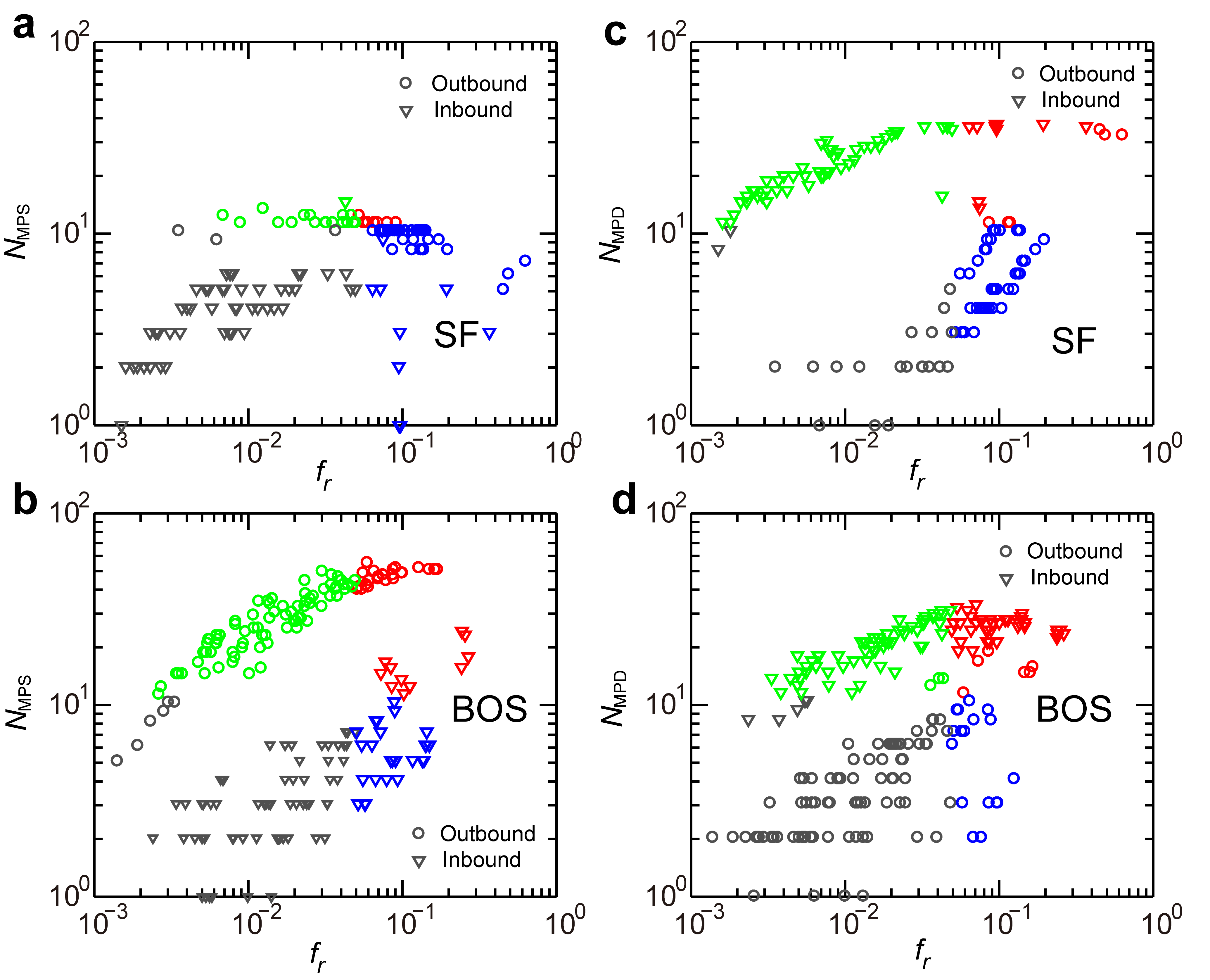

Supplement: Figure S2 — Correlations among , , and . (a) Four groups of URT segments were classified according to their and values. Red symbols represent the highly vulnerable segments () which also showed large numbers of major passenger sources . Blue symbols represent URT segments with high and low values, green symbols represent those with low and high values, and gray symbols represent those with low and low values. Circles represent the inbound URT segments, and triangles represent outbound URT segments. (b) The same classification system and symbols were used for Boston. (c) Red symbols represent URT segments with high and high values, Blue symbols represent URT segments with high and low values, green symbols represent those with low and high values, and gray symbols represent those with low and low values. (d) See (c). (TIF) [file pone.0080178.s002.tif]

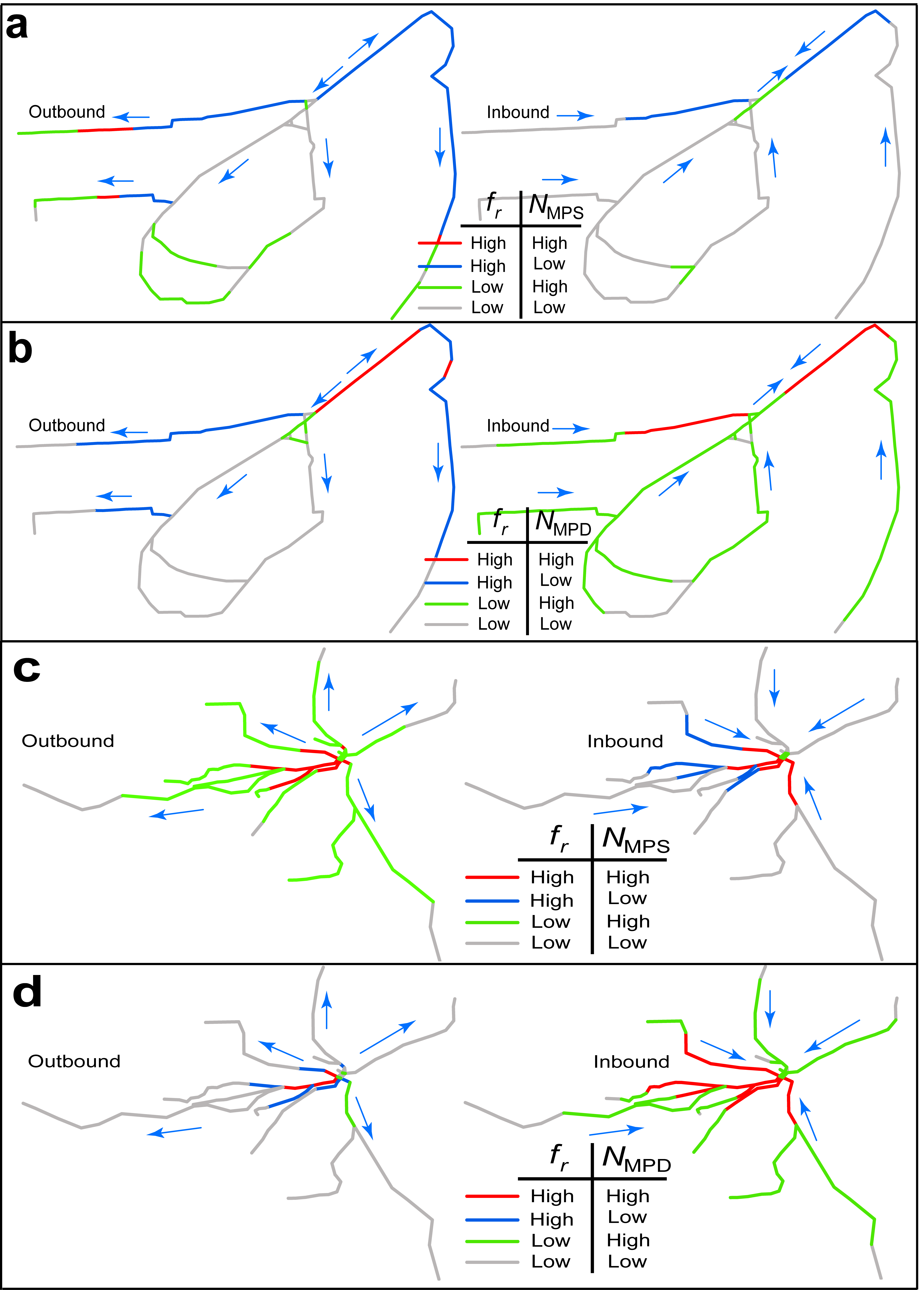

Supplement: Figure S3 — Spatial distributions of the eight groups of URT segments defined by , , and . (a) Spatial distribution of the four groups of San Francisco URT segments classified using to their and values. (b) The spatial distributions of the four groups of San Francisco URT segments were classified according to their and values. (c) Spatial distribution of the four groups of Boston URT segments classified using to their and values. (d) The spatial distributions of the four groups of Boston URT segments were classified according to their and values. (TIF) [file pone.0080178.s003.tif]
